# Comparative analysis of real-coded genetic algorithms for mixture distribution models: Insights from TOPSIS

**DOI:** 10.1371/journal.pone.0324198

**Published:** 2025-06-03

**Authors:** Jalal- ud-Din, Ehtasham-ul- Haq, Mohammed M. A. Almazah, Mhassen E. E. Dalam, Ishfaq Ahmad

**Affiliations:** 1 Department of Mathematics and Statistics, International Islamic University, Islamabad, Pakistan; 2 Department of Mathematics and Statistics, Faculty of Basic and Applied Sciences, International Islamic University, Islamabad, Pakistan; 3 Department of Mathematics, College of Sciences and Arts (Muhyil), King Khalid University, Muhyil, Saudi Arabia; Ningbo University, CHINA

## Abstract

This particular study presents two novel parent-centric real-coded crossover operators, named mixture-based Gumbel crossover (MGGX) and mixture-based Rayleigh crossover (MRRX), to increase the efficiency of genetic algorithms (GAs) in tackling complex optimization problems. Conventional crossover operators often struggle in multimodal and extremely restricted situations and fail to find the ideal balance between exploration and exploitation. Proposed parent-centric real-coded crossover operators increase the precision and robustness of GAs, which is confirmed by empirical results on testing constrained and un-constrained benchmark functions having different complexity levels. MGGX parent-centric real-coded crossover operator performs best in 20 out of 36 mean values cases and achieves the lowest standard deviation values in 21 out of 36 cases. Likewise, to confirm the efficiency, robustness, and reliability of the proposed crossover operator the Quade test, Performance index (PI), and multi-criteria TOPSIS method are utilized.

## 1 Introduction

### 1.1 Motivation and purpose

Optimized solutions are highly desirable across various domains of today’s rapidly evolving world. The fields of science, particularly computer science, engineering, management science, and applied mathematics are expanding rapidly. We face challenging optimization problems due to the complexity and modality of the problems [1–3]. Problems in science and engineering research are crucial nowadays because technology is changing the world quickly. Many of these problems are complex global optimization problems with high dimensions search spaces [4]. As the problem’s dimension increases, the search space expands exponentially, finding these problems challenging to solve [5]. Classical and gradient-based optimization methods struggle to handle modern complex problems due to the curse of dimensionality. Additionally, gradient-based searches are often ineffective when local optima exist, as they rely heavily on the choice of initial point [6].

Population-based stochastic methods including genetic algorithms (GA), simulated annealing (SA), particle swarm optimization (PSO), and feature selection techniques have been extensively used to overcome these limitations [7,8]. Heuristic and meta-heuristic approaches are crucial in multi-objective hybrid flow shop scheduling problems (HFSP) with machine capability limitations and constrained waiting times [9]. The problem’s complex nature is tackled by developing solution approaches based on GA [10]. Heuristic methods aim to efficiently approximate solutions with minimal computational efforts. The approximate techniques known as “meta-heuristics” are derived from various ideas in classical heuristics, neural systems, biological evolution, artificial intelligence, natural phenomena, and statistical mechanics [11]. Meta-heuristics inspired by nature are devised to address optimization issues that stem from various physical or biological phenomena. The values used by these algorithms are generated at random. The population can be optimized for iterations by adding the best individual value from the current generation to the next generation of individuals. They can manage several local optima simultaneously and effectively handle higher dimensional global optimization problems. These qualities have made these algorithms well-liked and widely utilized in solving global optimization problems. The researchers used all of these in developing optimization strategies that can address optimization issues in the real world. The two stages of the entire search process are called exploration and exploitation. As much random search as possible is preferred during the exploration stage, where the algorithm must explore the search space considering variables. The exploitation stage is determined by evaluating the possible areas discovered during exploration. The exploitation phase is connected to the local search process. Striking a balance between exploration and exploitation remains one of the primary challenges in optimization [12].

### 1.2 Literature review

Normally existing methods fail to strike a balance between exploration and exploitation, especially in multimodal optimization and high-dimensional problems, despite the development of crossover and mutation operators to enhance the efficiency of real-coded GAs [13]. Still, there is a lack of resilient, real-coded crossover operators that can maintain solution stability, avoid premature convergence, and frequently obtain ideal solutions across diverse optimization scenarios. Conventional operators such as double Pareto crossover (DPX), simulated binary crossover (SBX), and Laplace crossover (LX) struggle with population diversity, premature convergence, and adaptation to varying optimization landscapes [14]. To address these challenges, this study introduces two novel parent-centric real-coded crossover operators mixture-based Gumbel crossover and mixture-based Rayleigh crossover (MGGX and MRRX), designed to enhance the balance between exploration and exploitation dynamically. The gaps are filled by this study’s inclusion of two new real-coded crossover operators.

### 1.3 Contribution and paper organization

We suggest the mixture distribution-based MGGX and MRRX crossover operators to improve balancing between exploration and exploitation.

(i) Theoretically, these operators are developed to adapt dynamically, which reduces the risk of premature convergence while ensuring efficient exploration of complex search spaces. By leveraging the properties of mixture distributions and Gumbel distribution, they enable diverse yet high-quality offspring generation, improving GA robustness across different levels of benchmark functions.
(ii) By testing on constrained and unconstrained benchmark functions, we assessed proposed operators and examined that in most cases, the proposed operator especially MGGX outperforms conventional operators in terms of stability and efficiency, attaining the lowest mean and standard deviation. The inclusion of proposed operators within GA provides a practical approach to handling real-world optimization problems requiring strong consistent solutions. Both approaches PI and TOPSIS further validate MGGX’s robustness and scalability, making it an effective option for challenging, high-dimensional optimization tasks.

The remainder of this paper is organized as follows: Section 2 presents previously used operators. Section 3 introduces proposed mixture-based real-coded crossover operators. Section 4 details the benchmark functions. Section 5 discusses computational results, while Section 6 concludes the study with key finding and future research directions.

## 2 Previously used operators

For comparison, we considered the three real-coded crossover operators previously used in the literature. Laplace crossover operator (LX) was first proposed by Deep and Thakur [15].

It is a Laplace distribution-based crossover operator that is self-parent-centric. The double Pareto probability distribution, a parent-centric operator, is the foundation of DPX (Thakur, 2014). The procedure has three steps for generating two offspring, $\delta =(\delta_{1},\delta_{2},\ldots ,\delta_{n})$ & $\zeta =(\zeta_{1},\zeta_{2},\ldots \zeta_{n})$ from parents $w^{\left(1\right)}=\left(w_{1}^{\left(1\right)},w_{2}^{\left(1\right)},w_{3}^{\left(1\right)},\ldots ,w_{n}^{\left(1\right)}\right)$ and $w^{\left(2\right)}=\left(w_{1}^{\left(2\right)},w_{2}^{\left(2\right)},w_{3}^{\left(2\right)},\ldots ,w_{n}^{\left(2\right)}\right)$. In the first step, it begins the procedure by generating a random number (r_i_) uniformly distributed between zero and one. After a random number is generated in the second step, we calculate $\beta_{i}$ by simply inverting the Laplace cumulative distribution function in LX and the double Pareto cumulative distribution function in DPX. In the end, offspring are generated with the help of $\beta_{i}$:


$$\delta_{i}=\frac{{(w}_{i}^{\left\{1\right\}}+w_{i}^{\left\{2\right\}})+\beta_{i}\left|w_{i}^{\left\{1\right\}}-w_{i}^{\left\{2\right\}}\right|}{2}$$ (1)

and


$$\zeta i=\frac{{(w}_{i}^{\left\{1\right\}}+w_{i}^{\left\{2\right\}})-\beta_{i}\left|w_{i}^{\left\{1\right\}}-w_{i}^{\left\{2\right\}}\right|}{2}$$ (2)

Real-coded crossover operator SBX has a special feature with binary transformation to continuous search space, it was first proposed by Deb and Agarwal [16]. Important steps are adopted for generating following offspring, i.e., $\delta =(\delta_{1},\delta_{2},\ldots ,\delta_{n}$) by parents $w^{\left(1\right)}=\left(w_{1}^{\left(1\right)},w_{2}^{\left(1\right)},w_{3}^{\left(1\right)},\ldots ,w_{n}^{\left(1\right)}\right)$ & $w^{\left(2\right)}=\left(w_{1}^{\left(2\right)},w_{2}^{\left(2\right)},w_{3}^{\left(2\right)},\ldots ,w_{n}^{\left(2\right)}\right)$ are these:

(a)  Starting with a uniform distribution, generate a random number r_i_ between zero and one.
(b)  In this important step we obtain a parameter $\beta_{i}$ from mathematical expression.

Thus, from two parents $w^{\left(1\right)}\&$ $w^{\left(2\right)}$ and an offspring $\delta =(\delta_{1},\delta_{2},\ldots ,\delta_{n}$ is produced in the Equation 3:


$$\delta_{i}=\frac{1}{2}\left({(w}_{i}^{\left\{1\right\}}+w_{i}^{\left\{2\right\}}{)-\beta }_{i}\left|w_{i}^{\left\{1\right\}}-w_{i}^{\left\{2\right\}}\right|\right)$$ (3)

### 2.1 Mutation operators

Population diversity decreases the chance of premature convergence [17,18]. In literature, several mutation operators are used in GA to increase population diversity. In this study, we used three popular mutation operators. Non-uniform mutation (NUM) is one of the most popular mutation operators, and its working mechanism was introduced by Michalewicz et al. [19]. Makinen et al. proposed the Makinen, Periaux, and Toivanen Mutation (MPTM) [20]. MPTM is mostly used in literature to solve multidisciplinary optimization problems. We also used the third mutation operator named power mutation (PM) proposed by Deep and Thakur [21], which is based on power distribution. In GA the mutation operator helps in searching new search spaces and solving complex optimization problems.

## 3 Proposed mixture-based real-coded crossover operators

Several areas of science and engineering directly employ finite mixture models. Indirect applications of mixture models consist of the empirical Bayes method, cluster analysis, latent structure models, modeling of prior densities, and nonparametric (kernel) density estimation [22]. Here in this particular section, we present two real-coded crossover operators (named MGGX and MRRX). The idea is used to generate real-coded crossover operators based on the mixture of Gumbel distribution (MGGX) and then a mixture of Raleigh distribution (MRRX).

### 3.1 Proposed mixture Gumbel distribution-based real-coded crossover operator

A wide range of scientific disciplines, including geology, insurance, meteorology, hydrology, and finance, have used the Gumbel distribution and its extensions. It is commonly known that the tails of this distribution are heavier than those of the normal distribution. Since the Gumbel distribution has heavy tails, it can be used to model extreme happenings. In a dataset, it captures the likelihood of rare and extreme values. In extreme value theory, the Gumbel distribution is frequently utilized, particularly when modeling the distribution of maxima or minima in a sample [23]. The probability density function (pdf) of the Gumbel distribution is as follows:


$$f(p)=\frac{1}{\eta }e^{\left[-\frac{p-\mu }{\eta }-e^{\left(-\frac{p-\mu }{\eta }\right)}\right]}$$ (4)

where the cumulative distribution function (cdf) of Gumbel is as shown in equation 5 and *μ* denotes the location parameter and the scale parameter is denoted by η:


$$F(p;\mu ,\eta )=e^{-e^{-\left(\frac{p-\mu }{\eta }\right)}}$$ (5)

Pdf and cdf for the proposed two-component mixture probability model using Gumbel distribution can be written as follows:


$$f(p)=\gamma_{1}f\left(p_{1}\right)+\gamma_{2}f\left(p_{2}\right)$$ (6)

where $\gamma_{1}>0$, $\gamma_{2}<1$ and $\gamma_{1}+\gamma_{2}=1$


$$f(p)=\gamma_{1}1/(\eta_{1})e^{\left[-\frac{p_{1}-\mu_{1}}{\eta_{1}}-e^{\left(-\frac{p_{1}-\mu_{1}}{\eta_{1}}\right)}\right]}+\gamma_{2}1/(\eta_{2})e^{\left[-\frac{p_{2}-\mu_{2}}{\eta_{2}}-e^{\left(-\frac{p_{2}-\mu_{2}}{\eta_{2}}\right)}\right]}$$ (7)

$$F(p)=\gamma_{1}F\left(p_{1}\right)+\gamma_{2}F(p_{2})$$ (8)

$$F(p)=\gamma_{1}e^{-e^{-\left(\frac{p_{1}-\mu_{1}}{\eta_{1}}\right)}}+\gamma_{2}e^{-e^{-\left(\frac{p_{2}-\mu_{2}}{\eta_{2}}\right)}}$$ (9)

The cumulative function of the proposed two-component mixture distribution as elaborated in equation 9, has scale parameters as represented by $\eta_{1}$ and $\eta_{2}$, and the location parameters as $\mu_{1}$ and $\mu_{2}$. Using a planned procedure, we get two offspring ${(o}_{1}^{\left(1\right)},o_{2}^{\left(1\right)},\cdots ,o_{n}^{\left(1\right)})$ and ${(o}_{1}^{\left(2\right)},o_{2}^{\left(2\right)},\cdots ,o_{n}^{\left(2\right)})$ from two-parent ${p^{\left(1\right)}=(p}_{1}^{\left(1\right)},p_{2}^{\left(1\right)},\cdots ,p_{n}^{\left(1\right)}$ and ${p^{\left(2\right)}=(p}_{1}^{\left(2\right)},p_{2}^{\left(2\right)},\cdots ,p_{n}^{\left(2\right)}$ by employing mixture-based crossover. The three steps procedure is as follows:

i) By utilizing a uniform distribution we generate a random number denoted by $u_{i}$.
ii) The parametric value, shown as $\beta_{p}$ is then obtained by inverting cdf and generating random numbers from a mixture probability distribution:

$$\beta_{p}=\left\{ \begin{array}{c} \gamma_{1}\left[\mu_{1}-\eta_{1}\log\left\{-\log u_{i}\right\}\right]+\gamma_{2}\left[\mu_{2}-\eta_{2}\log\left\{-\log u_{i}\right\}\right],u_{i}\leq 0.5\\ \gamma_{1}\left[\mu_{1}-\eta_{1}\log\left\{-\log{(1-u}_{i})\right\}\right]+\gamma_{2}\left[\mu_{2}-\eta_{2}\log\left\{-\log\left(1-u_{i}\right)\right\}\right],u_{i}>0.5 \end{array}\right.$$ (10)

iii) In the end, two offspring are generated:


$$o_{i}^{\left(1\right)}=\frac{{(p}_{i}^{\left(1\right)}+p_{i}^{\left(2\right)})+\beta_{p}\left|p_{i}^{\left(1\right)}-p_{i}^{\left(2\right)}\right|}{2}$$ (11)

$$o_{i}^{\left(2\right)}=\frac{{(p}_{i}^{\left(1\right)}+p_{i}^{\left(2\right)})-\beta_{p}\left|p_{i}^{\left(1\right)}-p_{i}^{\left(2\right)}\right|}{2}$$ (12)

where i=1,2,⋯,n and variable bounds are $o_{i}<o_{i}^{l}$ or $o_{i}<o_{i}^{u_{i}}$, offspring are assigned randomly generated values in the interval $\left\{o_{i}^{l},o_{i}^{u_{i}}\right\}$ if they fall outside of these limitations.

### 3.2 Proposed mixture Rayleigh distribution-based real-coded crossover operator

The Rayleigh distribution is a positively skewed continuous distribution, is also used in wireless communications and signal processing due to its ability to model the magnitude of two-dimensional vectors with independent identical normal components. This distribution was first introduced by Lord Rayleigh [24]. In GA, its adaptive step-size mechanism enhances exploration in the early stages and refines exploitation later, which reduces the chances of pre-mature convergence. This property makes it suitable for high-dimensional and multimodal optimization problems. In equation 13 pdf and in equation 14 cdf of Rayleigh distribution are expressed.


$$f(g)=\frac{g}{\sigma^{2}}e^{-\frac{g^{2}}{2\sigma^{2}}}$$ (13)

$$F(g)=1-e^{-\frac{g^{2}}{2\sigma^{2}}}$$ (14)

where sigma denotes the scale parameter.

The cdf of the two-component mixture probability model for Rayleigh distribution is as follows:


$$F(g)=\varphi_{1}F\left(g_{1}\right)+\varphi_{2}F(g_{2})$$ (15)

where $\varphi_{1}>0$, $\varphi_{2}<1$ and $\varphi_{1}+\varphi_{2}=1$


$$F(g)=\varphi_{1}-\varphi_{1}e^{-\frac{g_{1}^{2}}{2\sigma_{1}^{2}}}+\varphi_{2}-\varphi_{2}e^{-\frac{g_{2}^{2}}{2\sigma_{2}^{2}}}$$ (16)

A stepwise process is used to obtain two offspring that are ${(\tau }_{1}^{\left(1\right)},\tau_{2}^{\left(1\right)},\cdots ,\tau_{n}^{\left(1\right)})$ and ${(\tau }_{1}^{\left(2\right)},\tau_{2}^{\left(2\right)},\cdots ,\tau_{n}^{\left(2\right)})$ from two parents ${g^{\left(1\right)}=(g}_{1}^{\left(1\right)},g_{2}^{\left(1\right)},\cdots ,g_{n}^{\left(1\right)}$ and ${g^{\left(2\right)}=(g}_{1}^{\left(2\right)},g_{2}^{\left(2\right)},\cdots ,g_{n}^{\left(2\right)}$by using two-component mixture distribution crossover. Where the three-step process is as follows:

a) The uniform distribution is used to generate a random number $v_{i}$.
b) To obtain $\beta_{g}$ in the second step, the cumulative density function of the Rayleigh distribution is inverted to generate a random number from the two-component mixture distribution.

$$\beta_{g}=\left\{ \begin{array}{c} \varphi_{1}\left[\sigma_{1}[2\log\left\{1-v_{i}\right\}{]}^{\frac{1}{2}}\right]+\varphi_{2}\left[\sigma_{2}[2\log\left\{1-v_{i}\right\}{]}^{\frac{1}{2}}\right],v_{i}\leq 0.5\\ \varphi_{1}\left[\sigma_{1}[2\log\left\{v_{i}\right\}{]}^{\frac{1}{2}}\right]+\varphi_{2}\left[\sigma_{2}[2\log\left\{v_{i}\right\}{]}^{\frac{1}{2}}\right],v_{i}>0.5 \end{array}\right.$$ (17)

c) The last step is the generation of two offspring for i=1,2,⋯,n:

$$\tau_{i}^{\left(1\right)}=\frac{{(g}_{i}^{\left(1\right)}+g_{i}^{\left(2\right)})+\beta_{g}\left|g_{i}^{\left(1\right)}-g_{i}^{\left(2\right)}\right|}{2}$$ (18)

$$\tau_{i}^{\left(2\right)}=\frac{{(g}_{i}^{\left(1\right)}+g_{i}^{\left(2\right)})-\beta_{g}\left|g_{i}^{\left(1\right)}-g_{i}^{\left(2\right)}\right|}{2}$$ (19)

where variable boundaries are $\tau_{i}<\tau_{i}^{l}$ or $\tau_{i}<\tau_{i}^{v_{i}}$, if offspring are outside these limits then randomly generated values $v_{i}$ are assigned to offspring $\tau_{i}$ in an interval $\left\{\tau_{i}^{l},\tau_{i}^{v_{i}}\right\}$.

## 4 Benchmark functions

Different types of optimization problems exist, varying in complexity and compatibility. Since there are no set criteria for choosing benchmark functions, there are many different kinds of benchmark functions with distinct characteristics in the literature. For this specific simulated-based study, we choose a set of twelve benchmark functions with various levels of compatibility and complexity. By using the proposed real-coded probabilistic-based algorithms on a set of twelve benchmark functions, we compare their effectiveness with current algorithms. These benchmark functions are extensively employed in numerous comparative analyses to verify the efficacy of algorithms the detail is given in Table 1. We take the first six unconstrained and the remaining six benchmark functions are constrained test problems from CEC 2017.

**Table 1:** Detail of benchmark functions comparison.

Sr. #	Test problem	Objective function	Limits
1	Levy and Montalvo-2 function	$\min_{x}f(x)=0.1\left(\sin^{2}(3\pi x_{1}\right)+\sum_{i=1}^{n-1}(x_{i}-1{)}^{2}\left[1+\sin^{2}\left(3\pi x_{i+1}\right)\right]+\left(x_{n}-1{)}^{2}\left[1+\sin^{2}\left(2\pi x_{n}\right)\right]\right)$	{-5, 5}
2	Neumair function	$\min_{x}f(x)=\sum_{i}^{n}(x_{i}-1{)}^{2}+\sum_{i=1}^{n-1}x_{i}x_{i-1}$	{-n^2^, n^2^}
3	Griewank function	$\min_{x}f(x)=1+\frac{1}{4000}\sum_{i=1}^{n}x_{i}^{2}-\prod_{i=1}^{n}cos(\frac{x_{i}}{\sqrt{i}})$	{-10, 10}
4	Brown3 function	$\min_{x}f(x)=\sum_{i=1}^{n-1}[{(x}_{i}^{2}hat{(x}_{i}^{2}+1)+(x_{i}+1^{2}){(x}_{i+1}^{2}+1)]$	{-1, 4}
5	Ellipsoidal function	$\min_{x}f(x)=\sum_{i=1}^{n}(x_{i}-i{)}^{2}$	{-n, n}
6	Cigar function	$\min_{x}f(x)=x_{1}^{2}+10000000\sum_{i=2}^{n}x_{i}^{2}$	{-10, 10}
7	C-01	$Minf(x)=\sum_{i=1}^{D}\left(\sum_{j=1}^{i}z_{j}\right)$^2^, z=x-0 $g(x=\sum_{i=1}^{D}\{z_{i}^{2}-5000\cos(0.1\pi z_{i})-4000\}\leq 0$	x∊{−100,100}^D^
8	C-02	$Minf(x)=\sum_{i=1}^{D}\left(\sum_{j=1}^{i}z_{j}\right)$^2^, z = x − 0, y = M * z $g(x=\sum_{i=1}^{D}\{y_{i}^{2}-5000\cos(0.1\pi y_{i})-4000\}\leq 0$	x {−100,100}^D^
9	C-03	$Minf(x)=\sum_{i=1}^{D}\left(\sum_{j=1}^{i}z_{j}\right)$^2^, z = x − o $g(x=\sum_{i=1}^{D}\{z_{i}^{2}-5000\cos(0.1\pi z_{i})-4000\}\leq 0$ h(x) = -$\sum_{i=1}^{D}z_{i}\sin\left(0.1\pi z_{i}\right)=0$	x {−100,100}^D^
10	C-04	$Minf(x)=\sum_{i=1}^{D}\{z_{i}^{2}-10\cos(2\pi z_{i})+10\}$,z = x − 0 $g_{1}(x=-\sum_{i=1}^{D}z_{i}sin(2z_{i})\leq 0$ $g_{2}(x=-\sum_{i=1}^{D}z_{i}sin(z_{i})\leq 0$	x {−10,10}^D^
11	C-05	$Minf(x)=\sum_{i=1}^{D}({100(z_{i}^{2}-z}_{i+1}{)}^{2}-(z_{i}-1{)}^{2})$,z = x − 0, y = M_1_ * z, w = M_2_ * z $g_{1}(x=\sum_{i=1}^{D}\{y_{i}^{2}-50\cos(2\pi y_{i})-40\}\leq 0$ $g_{2}(x=\sum_{i=1}^{D}\{w_{i}^{2}-50\cos(2\pi w_{i})-40\}\leq 0$	x {−10,10}^D^
12	C-06	$Minf(x)=\sum_{i=1}^{D}\{z_{i}^{2}-10\cos(2\pi z_{i})+10\}$, z = x − 0 $h_{1}(x=-\sum_{i=1}^{D}z_{i}sin(z_{i})=0$, $h_{2}(x=\sum_{i=1}^{D}z_{i}sin(z_{i})=0$, $h_{3}(x)=-\sum_{i=1}^{D}z_{i}cos(z_{i}=0$, $h_{4}(x)=\sum_{i=1}^{D}z_{i}cos(z_{i})=0$, $h_{5}(x=\sum_{i=1}^{D}(z_{i}sin(2*\sqrt{\left|z_{i}\right|}))=0$, $h_{6}(x=-\sum_{i=1}^{D}(z_{i}sin(2*\sqrt{\left|z_{i}\right|}))=0$	x ∊{−20,20}^D^

## 5 Computational results

### 5.1 The experimental framework

**Fig 1:**
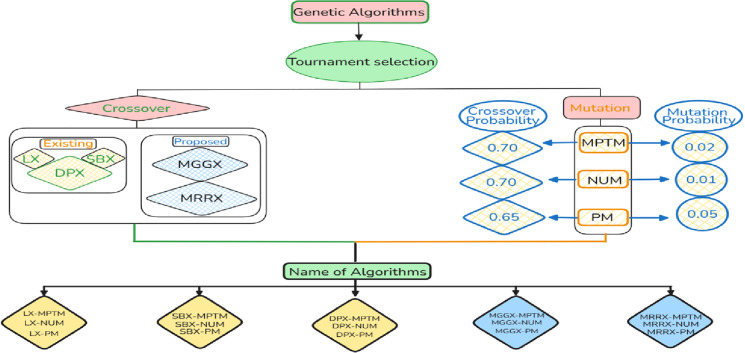
Visual parametric framework for all fifteen algorithms.

MGGX and MRRX are two proposed real-coded crossover operators compared to DPX, SBX, and LX three conventional crossover operators in this particular simulation-based study for global optimization tasks. So total of fifteen algorithmic combinations are produced by integrating these five crossover operators with three mutation operators which are NUM, MPTM, and PM. We test them on a set of twelve benchmark functions having both constrained and unconstrained scenarios. This set of benchmark functions was chosen for their diverse levels of modality and complexity. To retain the best individual for each generation, tournament selection with an elitism size of one was used for each of the fifteen algorithmic combinations. The termination criteria for all algorithmic combinations were fixed as five hundred generations, and the population size was also specified as three hundred. In Fig 1 we describe the specific mutation and crossover probabilities for each case.

### 5.2 Results and discussion

The goal is to evaluate the effectiveness of the suggested crossover operators with the simulation results over existing operators. We accomplish this by proposing a pair of new real-coded Probabilistic-based crossover operators one is the mixture-based real-coded crossover operator by using Gumbel distribution, and in the other operator, Rayleigh distribution is used. The following are the proposed mixture-based real-coded crossover operators MGGX-NUM, MGGX-MPTM, MGGX-PM, MRRX-NUM, MRRX-MPTM, and MRRX-PM. Table 2 shows the mean values of twelve benchmark functions having diverse characteristics for fifteen algorithms and similarly, Table 3 indicates the values of standard deviation of different benchmark functions. For multiple mutations (NUM, MPTM, and PM), Tables 2 and 3 demonstrate that the MGGX crossover outperforms all other operators. The computational complexity of the proposed approach is influenced by the mixture-based crossover operators. As shown in Table 4, proposed operators have a higher average execution time due to additional sampling and probabilistic combinations of distributions. The increase in run time is justified by better solution quality, robustness, and a better balance between exploration and exploitation in complex situations.

**Table 2:** Mean values of different benchmark functions.

Sr No.	Algorithm Names	Name of test problems/benchmark functions											
		LevyMont	Neumair	Griewank	Brown	Ellipsoidal	Cigar	C-01	C-02	C-03	C-04	C-05	C-06
**1**	**MGGX-NUM**	2.13E-05	4648.5	0.000725	0.0013	1.2299	4431	57000	53942	57129	188.147	55632	2540
**2**	**MRRX-NUM**	0.0057	20569	0.1153	0.2969	971.0723	2484800	54915	58231	57079	192.944	56908	2478.5
**3**	**DPX-NUM**	8.74E-05	3539.3	0.0016	0.0033	8.9061	27657	55562	56685	56448	192.034	55029	2650
**4**	**SBX-NUM**	0.0087	35852	0.2108	0.3586	591.2497	4612800	56653	56959	56195	185.841	57788	2528.5
**5**	**LX-NUM**	0.00014	8846.9	0.0024	0.004	3.3488	30023	55708	56354	56279	187.742	58183	2532.1
**6**	**MGGX-MPTM**	5.32E-06	260	6.58E-05	4.61E-05	0.856	765	56500	54600	54700	192	57402	2568.5
**7**	**MRRX-MPTM**	0.000852	2750	0.011	0.0148	1020	168000	55000	56000	57200	189.885	55882	2507.6
**8**	**DPX-MPTM**	1.81E-05	892.7273	0.000319	0.000362	8.0844	3640	55989	56841	55434	190.906	57216	2523.9
**9**	**SBX-MPTM**	0.0011	3060	0.0135	0.0153	616.77	262000	56297	56297	55951	192.934	57496	2570.5
**10**	**LX-MPTM**	2.06E-05	540.0498	0.000329	0.000184	4.9484	4210	56800	56484	55495	190.727	54485	2568.9
**11**	**MGGX-PM**	0.000102	7070	0.0025	0.0046	3.6	42400	56900	55400	56100	194	57686	2549.1
**12**	**MRRX-PM**	0.0061	22300	0.119	0.365	989	2640000	55200	55700	57500	192.651	57332	2570.7
**13**	**DPX-PM**	0.00045	5830	0.0076	0.0182	27.0527	122000	55481	56304	55776	190.215	56522	2545.2
**14**	**SBX-PM**	0.01	48200	0.22	0.4092	582.962	5180000	55244	56318	55873	189.8	57026	2524.7
**15**	**LX-PM**	0.000292	17700	0.011	0.0171	12.8189	147000	55700	56181	56066	190.618	55585	2527.7

**Table 3:** Stander deviation values of different benchmark functions.

Sr No	Algorithm Names	Name of test problems/benchmark functions											
		LevyMont	Neumair	Griewank	Brown	Ellipsoidal	Cigar	C-01	C-02	C-03	C-04	C-05	C-06
**1**	**MGGX-NUM**	1.43E-05	3340	0.0015	0.001	1.1764	2930	5150	6075	4436.9	14.5254	5406.9	219.789
**2**	**MRRX-NUM**	0.0024	7890	0.0354	0.0835	106.8213	727000	5498.9	4842.8	4544.8	11.1849	4506.4	213.631
**3**	**DPX-NUM**	5.59E-05	1250	0.000841	0.0022	3.038	11500	5404.4	4768.6	4917.4	11.9834	6233.5	199.509
**4**	**SBX-NUM**	0.0033	13900	0.0652	0.1069	92.7802	1320000	4381.7	6199.2	5449.7	17.4096	5500.1	212.476
**5**	**LX-NUM**	0.000113	4940	0.0017	0.003	1.9276	22500	4178.6	5468.3	4625.7	12.664	4938.8	226.017
**6**	**MGGX-MPTM**	1.07E-05	277	8.48E-05	4.96E-05	0.5486	690	5994.5	4828.2	5240.6	12.0147	4472	220.584
**7**	**MRRX-MPTM**	0.000779	3110	0.0141	0.0126	109.6718	144000	6052.7	5389.3	5377	13.1197	5344.4	256.564
**8**	**DPX-MPTM**	2.28E-05	1060	0.00061	0.000359	6.2504	3480	5389.3	4765.8	4769.4	14.4426	5717.4	212.472
**9**	**SBX-MPTM**	0.000765	2640	0.0166	0.0093	88.5754	351000	6526.6	6526.6	5675.2	16.7739	5017.1	219.754
**10**	**LX-MPTM**	3.61E-05	666.967	0.000708	0.000211	4.6375	4300	4765.8	5994.5	5169.1	18.8	6750.9	224.024
**11**	**MGGX-PM**	5.46E-05	5210	0.0015	0.0044	2.744	62300	4699.9	5249.7	4253.5	9.3703	5309.4	191.608
**12**	**MRRX-PM**	0.0018	7070	0.0321	0.0843	90.2339	833000	4709.3	4162.9	5315.2	16.5521	5214.5	199.698
**13**	**DPX-PM**	0.000248	1990	0.0032	0.015	12.8489	91600	5475.6	5038.1	6052.6	13.5575	6635.7	222.265
**14**	**SBX-PM**	0.0036	17500	0.0643	0.1433	87.0768	1860000	3894.6	5000.6	6033.5	13.9061	5572.5	229.488
**15**	**LX-PM**	0.000284	10600	0.0104	0.0157	9.5745	121000	5336.5	4480.1	4541.2	16.9681	6653.9	203.504

**Table 4:** Average execution time (in seconds) of different benchmark functions.

Sr No	Algorithm Names	Name of test problems/benchmark functions											
		LevyMont	Neumair	Griewank	Brown	Ellipsoidal	Cigar	C-01	C-02	C-03	C-04	C-05	C-06
**1**	**MGGX-NUM**	250.520	149.192	219.588	332.032	327.211	273.168	312.686	246.703	305.385	269.575	307.956	196.841
**2**	**MRRX-NUM**	91.070	79.411	117.301	156.226	109.449	177.686	95.771	88.409	107.130	84.111	80.512	143.794
**3**	**DPX-NUM**	83.698	130.145	82.579	341.124	96.815	87.860	68.933	86.584	87.314	81.907	93.998	155.427
**4**	**SBX-NUM**	78.645	141.177	80.651	165.874	88.736	181.107	81.769	121.203	87.363	84.620	90.455	186.625
**5**	**LX-NUM**	107.713	181.630	81.458	166.843	96.063	106.539	82.792	71.674	86.876	81.813	118.607	114.005
**6**	**MGGX-MPTM**	238.793	258.620	279.197	248.384	182.391	237.992	252.858	178.038	278.261	254.664	313.631	210.098
**7**	**MRRX-MPTM**	85.230	122.743	84.781	135.553	149.816	92.471	60.464	96.763	86.370	102.885	90.785	89.472
**8**	**DPX-MPTM**	104.785	92.002	93.857	189.796	97.119	75.669	74.046	107.380	106.126	98.964	98.727	182.161
**9**	**SBX-MPTM**	102.961	87.965	87.568	131.532	89.222	75.015	112.032	87.744	118.770	126.240	112.848	111.366
**10**	**LX-MPTM**	102.363	82.434	94.865	161.537	92.945	72.342	88.857	118.286	128.817	78.854	126.841	113.766
**11**	**MGGX-PM**	226.266	170.237	250.553	206.320	208.220	246.831	166.323	155.558	256.929	205.197	187.798	144.809
**12**	**MRRX-PM**	89.740	108.791	126.972	129.436	87.211	85.800	72.960	98.425	77.687	73.454	97.676	124.794
**13**	**DPX-PM**	87.889	77.274	79.575	130.299	87.473	102.063	76.170	125.256	92.763	75.746	61.385	101.608
**14**	**SBX-PM**	84.798	74.559	73.456	141.748	83.054	94.860	106.832	68.037	82.136	70.675	62.621	119.214
**15**	**LX-PM**	84.625	76.388	77.445	118.098	81.374	103.471	92.151	101.134	94.906	82.980	62.129	77.007

### 5.3 Performance index

It is common practice to compare different heuristic algorithms utilizing the performance index. It examines the behavior of various stochastic search strategies. Performance index (PI) is used here to obtain a more comprehensive understanding of the respective performance of MGGX-NUM, MGGX-MPTM, MGGX-PM, MRRX-NUM, MRRX-MPTM, and MRRX-PM algorithms, this performance index was also used in literature [18]. The least mean and least standard deviation are given prescribed weighted importance by this index. Equation 20 provides a mathematical setup of this PI.


$$PI=\frac{1}{N_{p}}\sum_{i=1}^{N_{p}}\left(b_{1}\beta_{1}^{i}+b_{2}\beta_{2}^{i}+b_{3}\beta_{3}^{i}\right)$$ (20)

where


$$\beta_{1}^{i}=\frac{{Mean}^{i}}{{mean}^{i}}$$

$$\beta_{2}^{i}=\frac{{Sd}^{i}}{{sd}^{i}}$$

$$\beta_{3}^{i}=\frac{{CV}^{i}}{{cv}^{i}}fori=1,2,3,\ldots ,N_{p}$$

and *Np* denotes the total number of populations, ${sd}^{i}$ denotes the least standard deviation and ${CV}^{i}$ denotes the least value of the coefficient of variation. Where$b_{1}$,$b_{2}$, and $b_{3}$ denote weights and the sum of weights equal to one. Following is the mathematical setup of different three cases.

1^st^ case $b_{1}=weight$, $b_{2}=b_{3}=\frac{1}{2}(1-weight)$
2^nd^ case $b_{2}=weight$, $b_{1}=b_{3}=\frac{1}{2}(1-weight)$
3^rd^ case $b_{3}=weight$, $b_{1}=b_{2}=\frac{1}{2}(1-weight)$

And for all three cases, weights are between 0 and 1.

We also employed the Quade test, a non-parametric test that adopts ranking methodology [18], to compare the performance of two proposed and three conventional real-coded crossover operators. Here equation number 21 represents the mathematical formulation of the Quade test statistics.


$$Q_{teststatistic}=\frac{(b-1)\sum_{j=1}^{p}w_{j}^{2}}{b\sum_{i=1}^{b}\sum_{j=1}^{p}w_{ij}^{2}-\sum_{j}^{p}w_{j}^{2}}$$ (21)

where,

$w_{ij}^{2}=k_{i}(R_{ij}-\frac{\left(b+1\right)}{2})$, $w_{j}=\sum_{i=1}^{b}w_{ij}$, p = number of crossover operator, b = number of mutation operator, and $k_{i}$ denotes ranked range.

The Quade test statistics value is 5.65 having a p-value of 0.004 concluding a significant difference between crossover operators (MGGX, MRRX, DPX, SBX, and LX). The pairwise comparison is represented in Table 5 below. It demonstrates strong evidence that the proposed operator MGGX outperforms SBX, DPX, MRRX, and LX.

**Table 5:** Comparison of different crossover operators.

Pairs of crossover operators	P-value
MGGX vs SBX	0.0003
MGGX vs DPX	0.0091
MGGX vs MRRX	0.0096
MGGX vs LX	0.0149

**Fig 2:**
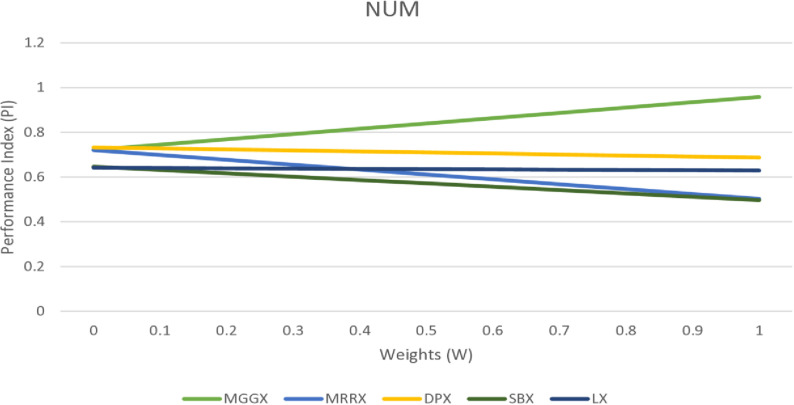
Visual line representation of algorithms with NUM mutation.

**Fig 3:**
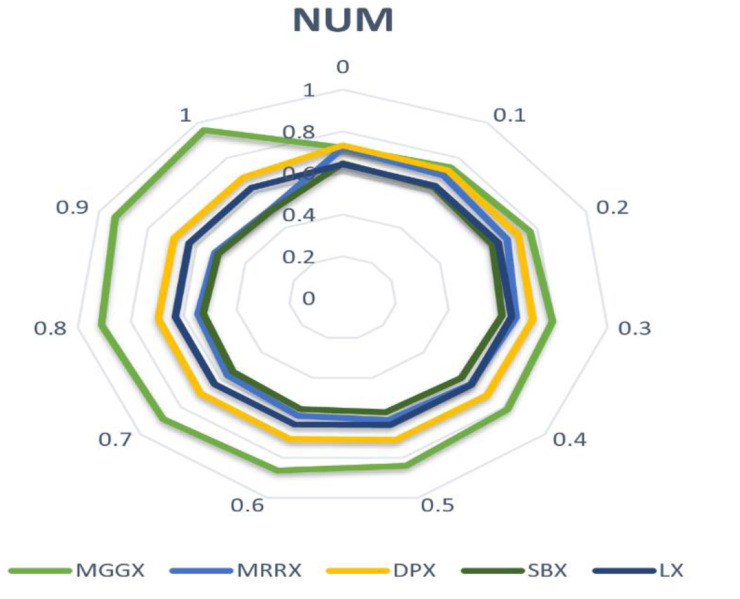
Visual line representation of algorithm with MPTM mutation.

**Fig 4:**
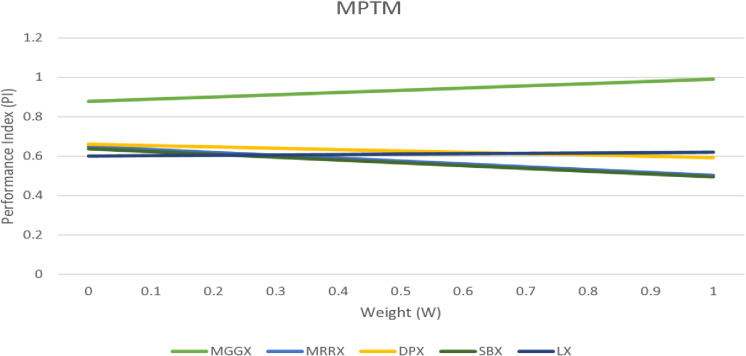
Visual line representation of algorithm with PM mutation.

**Fig 5:**
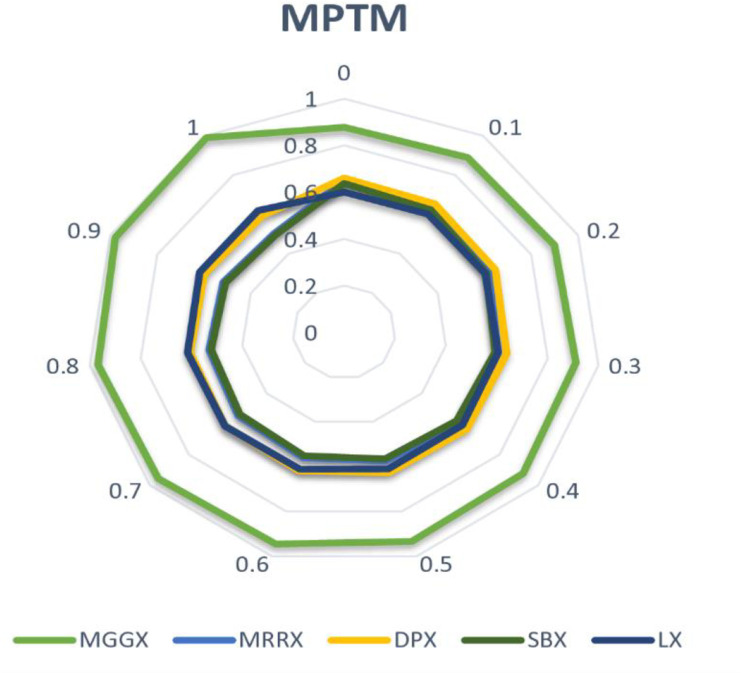
Visual performance of each algorithm with NUM mutation.

**Fig 6:**
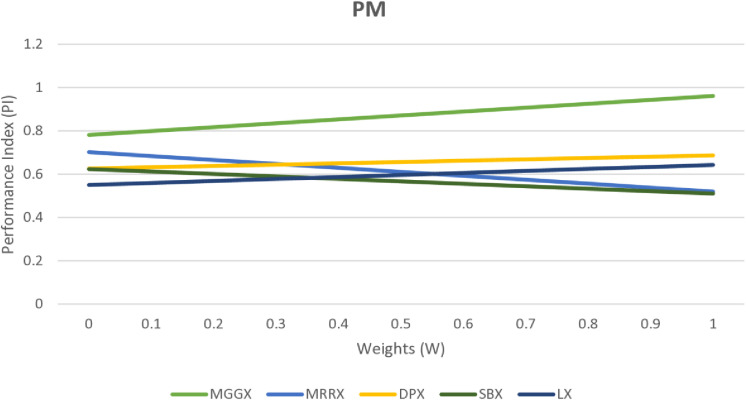
Visual performance of each algorithm with MPTM mutation.

**Fig 7:**
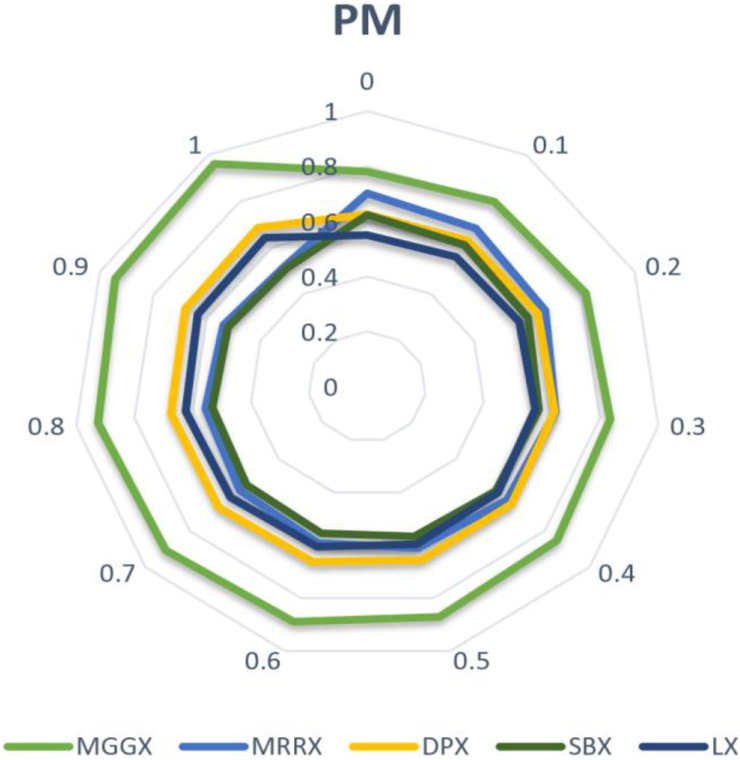
Visual performance of each algorithm with PM mutation.

In Figs 2, 3, and 4 line graph shows that MGGX is consistently outperforming all other real-coded operators, showing that the MGGX crossover operator is outperforming the others in the form of a plotted metric line. This growing pattern suggests that the proposed crossover operator (MGGX) is approaching the global solution. As the algorithm develops its search, a more successful global solution exploration is made possible by the innovative crossover operator (MGGX). Visual performance of each algorithm with mutations NUM, MPTM, and PM are expressed in Figs 5, 6, and 7 respectively, MGGX performs outperforming in all three cases.

### 5.4 TOPSIS multi-criteria decision-making method

One common approach to solving multiple objective decision-making problems is to identify compromise solutions for each of the multiple objective functions. People generally want to make optimal decisions when faced with several options [25]. Developing analytical and numerical techniques that consider multiple alternatives with multiple criteria is the goal, of using scientific terminology. TOPSIS (Technique for Order Preference by Similarity to Ideal Solution) is a numerical method for multi-criteria decision-making [26]. This method is broadly applicable and makes use of a straightforward mathematical framework. It is also an effective strategy that requires technological support. The method has been applied to optimal decisions throughout the last few decades.

**Fig 8:**
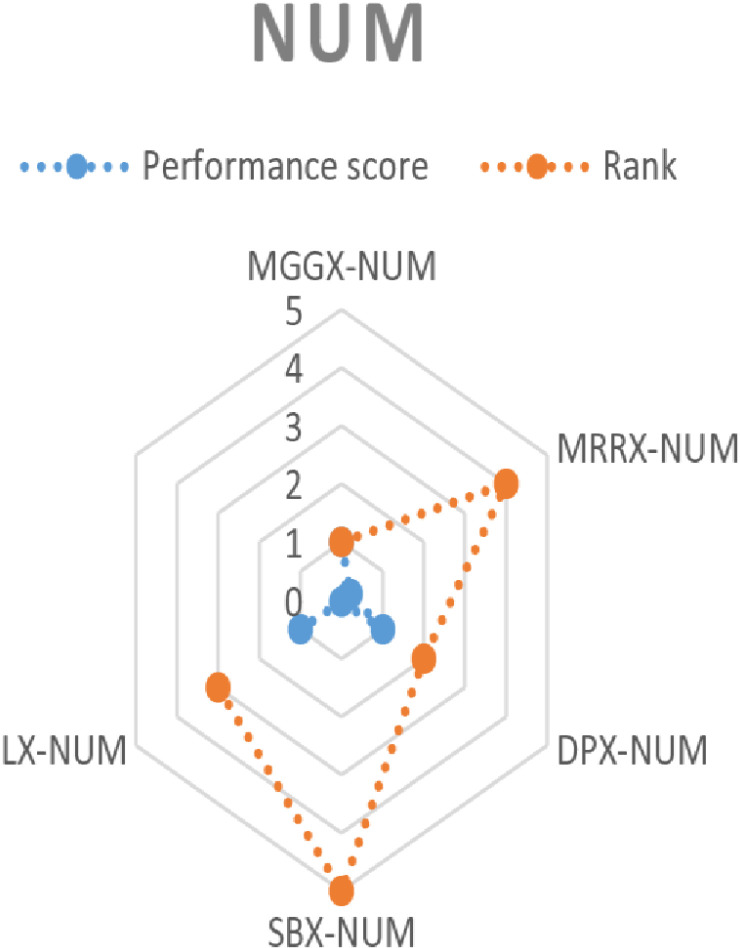
Visual representation of ranks of algorithms with NUM mutation.

**Fig 9:**
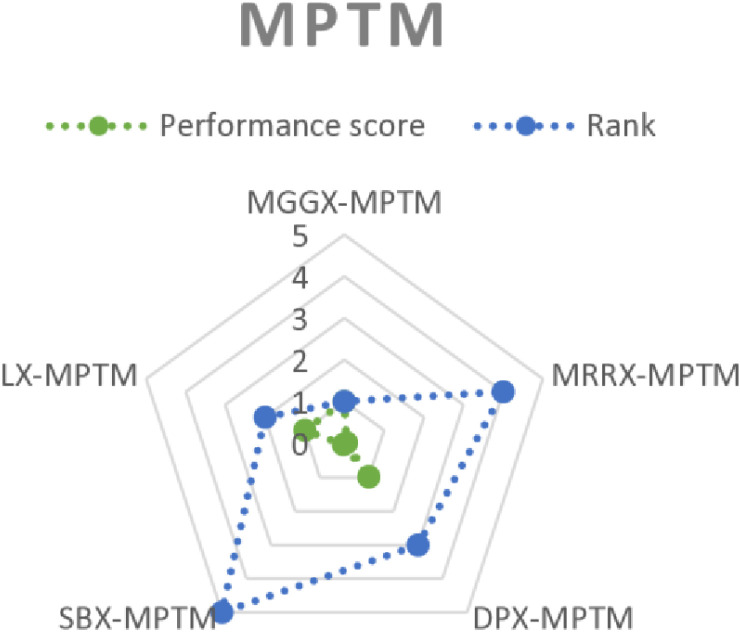
Visual representation of ranks of algorithms with MPTM mutation.

**Fig 10:**
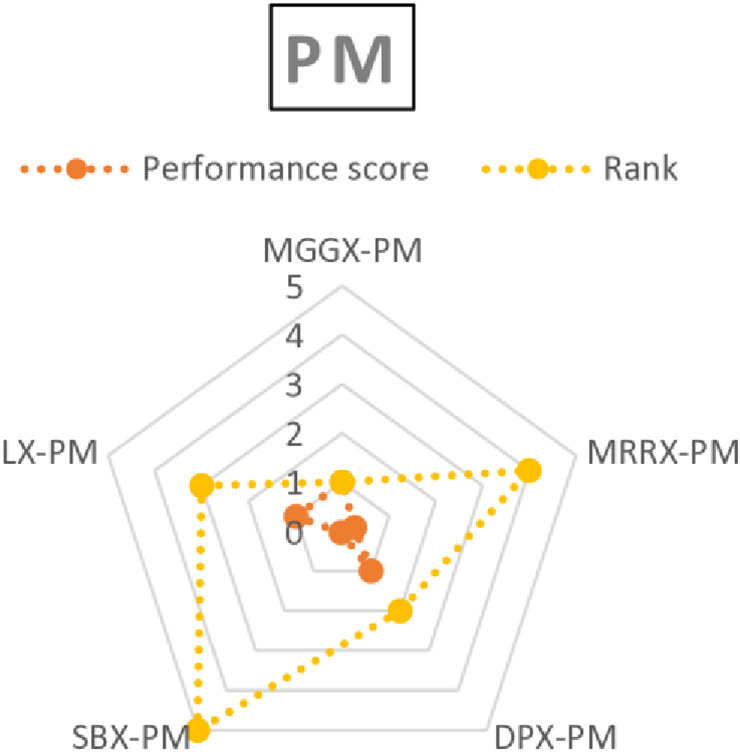
Visual representation of ranks of algorithms with PM mutation.

Based on the performance score of the TOPSIS, a rank was established for every algorithm as shown in Tables 6–8. Consequently, MGGX surpassed the two closest competitors, DPX and LX, to become the most preferred algorithm for every mutation operator among all of these options. Figs 8–10’s visual representation of the ranks for each algorithm further demonstrates how well the recently suggested MGGX algorithm performs.

**Table 6:** The ranking of algorithms with NUM mutation by TOPSIS.

Name of algorithms	Ideal Best	Ideal Worst	Performance score	Rank
**MGGX-NUM**	8.17E-06	0.02838385	0.99971223	1
**MRR-NUM**	0.0141748	0.00384161	0.2132288	4
**DPX-NUM**	1.223E-05	0.02834789	0.99956871	2
**SBX-NUM**	0.0254645	0.00078134	0.0297702	5
**LX-NUM**	0.000117	0.02708503	0.9956998	3

Source: author’s work.

**Table 7:** The ranking of algorithms with MPTM mutation by TOPSIS.

Name of algorithms	Ideal Best	Ideal Worst	Performance score	Rank
**MGGX-MPTM**	5.656E-06	0.0249744	0.999773567	1
**MRR-MPTM**	0.0181073	0.0010416	0.054395124	4
**DPX-MPTM**	0.0001644	0.0231858	0.992958897	3
**SBX-MPTM**	0.021755	0.0007956	0.035280368	5
**LX-MPTM**	3.788E-05	0.0238903	0.998417062	2

Source: author’s work.

**Table 8:** The ranking of algorithms with PM mutation by TOPSIS.

Name of algorithms	Ideal Best	Ideal Worst	Performance score	Rank
**MGGX-PM**	7.35E-06	0.028137546	0.999738791	1
**MRRX-PM**	0.01339874	0.004715359	0.26031431	4
**DPX-PM**	1.8325E-05	0.027122303	0.999324796	2
**SBX-PM**	0.02501556	0.000871786	0.033676167	5
**LX-PM**	0.00032063	0.025364846	0.987516989	3

Source: author’s work.

## 6 Conclusions and future research

This study introduces a unique approach using the mixture distribution. A pair of new crossover operators are proposed using Gumbel and Rayleigh distributions separately and efficiency is compared with the three operators in the literature. To examine these five crossover operator versions (MGGX, MRRX, DPX, SBX, and LX) with the co-integration of three mutation operators (NUM, MPTM, and PM), twelve widely recognized benchmark functions with diverse characteristics are utilized. Real-coded algorithms of mixture distribution are designed by combining two components of Gumbel distribution with 3 mutations (MGGX-NUM, MGGX-MPTM, and MGGX-PM). Similarly, real-coded algorithms are proposed using Rayleigh distribution (MRRX-NUM, MRRX-MPTM, and MRRX-PM). The strategies are compared concerning the lowest mean objective function values and standard deviation values. Furthermore, this study employs the performance index (PI) and the multi-criteria decision-making technique TOPSIS for comparison. It is confirmed through these techniques (PI & TOPSIS) that proposed algorithms MGGX-NUM, MGGX-MPTM, and MGGX-PM perform significantly better than all the algorithms taken into consideration for comparison in the present case.

The proposed operators have a great deal of managerial potential for resolving challenging optimization problems in real-world situations. The flexibility of the MGGX operator to efficiently handle high-dimensional and multimodal search spaces could be useful for supply chain management, scheduling, and resource allocation applications. Decision-makers in fields like engineering, manufacturing, and logistics might use these insights to develop optimal solutions that are more resilient and need less computing power.

### 6.1 Limitations

Despite this study evaluating the proposed operators on both constrained and unconstrained benchmark functions, it focused merely at standard benchmark suites and did not evaluate the operators’ effectiveness for use in specific scenarios.

### 6.2 Future research directions

Future research could explore the application of MGGX and MRRX to practical problems, such as multimodal optimization in resource allocation, supply chain logistics, and scheduling. To enhance generalization, the operators will be tested on benchmark suites beyond CEC 2014/2015 and CEC 2017. Additionally, hybrid strategies incorporating adaptive parameter mechanisms and combining MGGX and MRRX with other meta-heuristics could further improve their performance and adaptability across various optimization scenarios.
